# [Retracted] MicroRNA‑336 directly targets Sox‑2 in osteosarcoma to inhibit tumorigenesis

**DOI:** 10.3892/mmr.2024.13264

**Published:** 2024-06-17

**Authors:** Yong Cao, Tianding Wu, Dongzhe Li, Jianzhong Hu, Hongbin Lu

Mol Med Rep 15: 4217–4224, 2017; DOI: 10.3892/mmr.2017.6493

Following the publication of this paper, it was drawn to the Editor's attention by a concerned reader that certain of the Transwell cell migration and invasion assay data in Fig. 3C and D, and the tumour images shown in Fig. 4A were strikingly similar to data appearing in different form in other articles written by different authors at different research institutes, which had already been published. In addition, certain of the data panels shown in Fig. 3C were overlapping, such that the data from the same original source had been selected to represent the results from allegedly differently performed experiments. Owing to the fact that the contentious data in the above article had already been published prior to its submission to *Molecular Medicine Reports*, the Editor has decided that this paper should be retracted from the Journal. The authors were asked for an explanation to account for these concerns, but the Editorial Office did not receive a reply. The Editor apologizes to the readership for any inconvenience caused.

